# Co-activation of rhythms during alpha band oscillations as an interictal biomarker of exploding head syndrome

**DOI:** 10.1177/0333102420902705

**Published:** 2020-04-10

**Authors:** Dimitris Fotis Sakellariou, Alexander David Nesbitt, Sean Higgins, Sandor Beniczky, Jan Rosenzweig, Panagis Drakatos, Nadia Gildeh, Patrick Brian Murphy, Brian Kent, Adrian John Williams, Meir Kryger, Peter J Goadsby, Guy Doron Leschziner, Ivana Rosenzweig

**Affiliations:** 1 Sleep and Brain Plasticity Centre, Department of Neuroimaging, Institute of Psychiatry, Psychology and Neuroscience (IoPPN), King’s College London, London, UK; 2 Basic and Clinical Neuroscience, IoPPN, King's College London, London, UK; 3 Sleep Disorders Centre, Guy’s and St Thomas’ NHS Foundation Trust, London, UK; 4 Headache Group, Department of Clinical Neurosciences, King’s College Hospital NHS Foundation Trust, London, UK; 5 Danish Epilepsy Centre, Dianalund, Denmark; 6 Aarhus University Hospital, Aarhus, Denmark; 7 Department of Engineering, King’s College London, UK; 8 Pulmonary, Critical Care and Sleep Medicine, Yale School of Medicine, Connecticut, USA; 9 NIHR-Wellcome Trust Clinical Research Facility, SLaM Biomedical Research Centre, King’s College London, London, UK

**Keywords:** Exploding head syndrome, sleep-wake transition, parasomnia, sleep, EEG, alpha rhythm

## Abstract

**Background:**

Exploding head syndrome is a rarely reported benign sensory parasomnia that may nonetheless have significant impact on patients’ quality of life and their perceived well-being. To date, the mechanisms underlying attacks, characterised by a painless perception of abrupt, loud noises at transitional sleep-wake or wake-sleep states, are by and large unclear.

**Methods and results:**

In order to address the current gap in the knowledge of potential underlying pathophysiology, a retrospective case-control study of polysomnographic recordings of patients presenting to a tertiary sleep disorders clinic with exploding head syndrome was conducted. Interictal (non-attack associated) electroencephalographic biomarkers were investigated by performing macrostructural and event-related dynamic spectral analyses of the whole-night EEG. In patients with exploding head syndrome, additional oscillatory activity was recorded during wakefulness and at sleep/wake periods. This activity differed in its frequency, topography and source from the alpha rhythm that it accompanied.

**Conclusion:**

Based on these preliminary findings, we hypothesise that at times of sleep-wake transition in patients with exploding head syndrome, aberrant attentional processing may lead to amplification and modulation of external sensory stimuli.

## Introduction

Exploding head syndrome (EHS) is an unusual paroxysmal sensory parasomnia (1), which is characterised by the painless perception of abrupt, loud noises at transitional sleep-wake or wake-sleep states (2,3). Goadsby and Sharpless have recently argued that the term exploding head syndrome is an incomplete description, and have proposed the use of the term Episodic Cranial Sensory Shock to more accurately reflect the original 1876 account given by Mitchell, in whose case report a patient with a notion of a “pistol shot” going off in the night, is described (4). A chronic occurrence of the attacks, with a highly variable frequency, from one attack every few days to several attacks per night, is common. A study of parasomnia prevalence, which applied the Munich Parasomnia Screening questionnaire to 180 healthy participants, found a lifetime prevalence of EHS of 11% (5), with a second, slightly larger and specific study of young adults yielding 18%, within which 16.5% reported experiencing recurrent episodes (6). Building on these population-based data, Denis and colleagues recently surveyed 199 female undergraduates to find a lifetime prevalence of 37.2% (6.5% experiencing monthly episodes), with insomnia and sleep paralysis showing a significant co-association with EHS (7). In a much larger international sample of 1683 participants, the same authors demonstrated an additionally high lifetime prevalence of 29.6% for EHS, with monthly episodes occurring in 3.9% (7). In this sample, in addition to insomnia and sleep paralysis, nightmares, and dissociative episodes during wakefulness, a co-association after application of multiple logistic regression models was also demonstrated (7).

The male-to-female ratio in the population-based studies described above, which considered both sexes, is roughly equal (5–7). This is in contrast to the notion that EHS typically affects older women, which stems from a meta-analysis of case-series data (8). There are no population-based data concerning peak ages of onset, but it has been reported in individuals as young as 12 and as old as 84 (9). The meta-analysis of published cases by Frese and colleagues suggested a median age of onset of 58 years, but this is likely to represent the increased referral rate of older adults reporting the symptoms as a result of anxiety surrounding age-related intracranial pathology (8). Sharpless noted in a population-based study of young adults that only 11% had sought medical attention for the episodes, and within this group, none consulted with a doctor who was familiar with the disorder (6). This observation was also noted by Pearce in his original case series (2). Anecdotal reports have suggested symptoms of EHS occurring in family members, but no pedigree studies are available to analyse potential modes of inheritance.

To date, no study has examined the natural history of the disorder. Some case reports have reported spontaneous resolution or loss to follow-up, suggesting the symptoms may be self-limiting or improve with age. From the data available, there do not appear to be any significant medical or psychiatric sequelae that EHS appears to be a precursor of, bar for insomnia (7).

Patients with EHS can need specialist referral, typically for reassurance, and often to a headache clinic. In addition, patients with EHS may frequently have a coexistent, often episodic, primary headache disorder. Migraine with aura, primary stabbing headache, chronic tension-type headache, primary exertional headache, and primary headache associated with sexual activity are all variably recorded in case reports (8,9). In addition, stress and anxiety have been proposed as important risk factors, and there is a suggestion that anxiolytic treatment may ameliorate the EHS symptoms (10). Despite its association with significant fear and distress, to date the pathophysiology of this sleep disorder is largely unclear (3,11). Over the years, several competing hypotheses have been put forward. Previous studies have argued that sudden involuntary movements of middle ear components or the Eustachian tube might underlie the distinct mechanism behind this phenomenon (2,3). Others have hypothesised the association of EHS with a delay of activity reduction in areas of the brainstem reticular formation during sleep initiation (3,11). Similarly, the overactive or aberrant activity in the occipital network has been theorised (10) and more recently, transient calcium channel dysfunction in patients with EHS has also been suggested as a possible mechanism (11). The aim of this study was to explore biomarkers of EHS by performing macrostructural and event-related dynamic spectral analysis of the whole-night electroencephalogram (EEG).

## Methods

A retrospective case-control study of polysomnographic recordings with EHS, who were investigated between 2015 and 2018 at a large tertiary Sleep Disorders Centre (Guy’s Hospital, London, United Kingdom), was conducted (12). The study obtained required ethical approval (Project No 9342, GSTT NHS).

More specifically, a database of all clinical diagnostic polysomnographic (PSG) recordings performed between 1 January 2015 and 31 December 2018 at the Sleep Disorders Centre, Guy’s Hospital (London, United Kingdom), was retrospectively searched for the term “exploding head syndrome” in the diagnostic coding section of the database. Once identified, the case notes of the individual patients were reviewed by two independent clinicians, certified in sleep medicine, to ensure they satisfied the diagnostic criteria for EHS, as defined by the ICSD-3 diagnostic criteria (American Academy of Sleep Medicine, 2014) (13). Thereafter, the individual patient records were reviewed, together with the clinical overnight diagnostic PSGs, to ensure no additional sleep disorders were present, and predominant over EHS in terms of their sleep complaints, and no overt neurological (with the exception of primary headache disorders) or psychiatric comorbidities existed. In addition, current prescription psychotropic, neuromodulatory and/or hypnotic drug use, as well as illicit drug use, led to exclusion.

Once the EHS cohort was defined, simple demographics (age, sex) and duration of Wakefulness After Sleep Onset (WASO) were used to select matched control subjects from a subset of patients entered into the same database with a 2:1 ratio. These subjects presented over the same time period with non-diagnostic sleep complaints (the search term used to mine the database was “unrefreshing sleep” entered as the patient’s presenting complaint), but unremarkable pre-admission sleep diaries and overnight PSG studies (unremarkable is defined as sleep efficiency > 80%; evidence of normal NREM-REM sleep cycling, and no evidence of sleep pathologies including sleep-disordered breathing, sleep-related movement disorders, non-rapid eye movement (REM) or REM parasomnias or wake-sleep-REM transitional instability). Once identified, case notes were reviewed by the independent clinician to ensure no overt neurological (with the exception of primary headache disorders), psychiatric or general medical comorbidities existed. The same pharmacological exclusion criteria used in the EHS group were also employed.

Manual sleep staging was performed by two independent sleep experts by visual inspection of the EEG recordings along with electrooculogram (EOG) and electromyography (EMG) channels. The criteria by the AASM Visual Scoring Task Force and the DGSM Task Force (14) were used, by using a display time resolution of one second. The latency to non-rapid eye movement (NREM) sleep stage N1 was measured until the occurrence of 30.0 seconds of consecutive N1 sleep stages. A rapid eye movement (REM) period was defined as lasting a minimum of 240.0 seconds (s), with a minimum duration of 60.0 s between REM periods. A slow wave sleep (SWS, or N3) period was also defined as lasting a minimum of 240.0 s, with a minimum duration of 60.0 s between SWS periods.

EEG records analysis was performed on the scalp sensor level. The whole-night Time-Frequency Analysis (TFA); that is, hypnospectrogram (15) was derived using short-time Fourier transformation (STFT) with a 4096-point window and 4068 overlap on the signal of O1, O2, C3 and C4 electrodes independently, for frequencies in the range 0.05–45 Hz at a step of 0.03 Hz. Similarly, spectral topography and event-related; that is, ACA-related TFA analysis, was estimated using event-related FFT-based transformations. The hypnospectrograms were investigated in context with the relevant hypnograms to identify the sleep stage that possibly outstanding spectral components occur in. The data was not filtered for the purpose of whole-night spectral EEG analysis.

Source localization was performed using a standard realistic head model, as previously described and as implemented in the software package ASA (http://www.ant-neuro.com). EEG records were band pass filtered to alpha (8.5–12.5 Hz) and theta (3.5–8.5 Hz) for the purpose of source localization (16) and current density was estimated on the cortical surface using the sLORETA (Standardized Low Resolution Electromagnetic Tomography) method of Pascual-Marqui (17) and Dale et al. (2000) (18); all sLORETA calculations were performed in the frequency domain.

## Results

**Figure 1.**
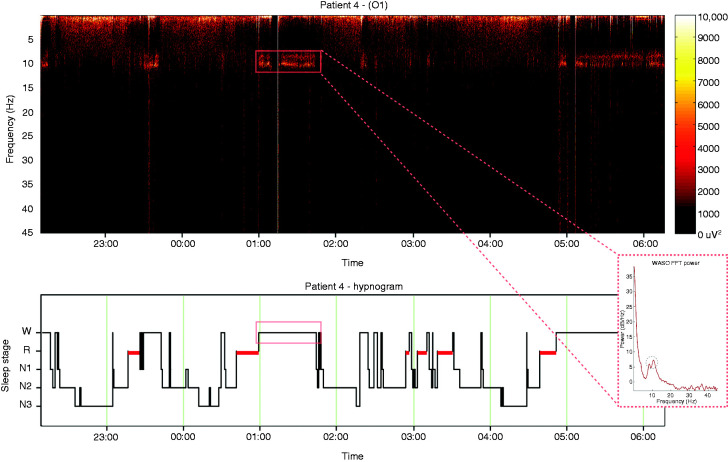
Representative whole-night EEG time-frequency analysis of a patient with exploding head syndrome derived using short-time Fourier transformation (top) and co-registered hypnogram (bottom). The FFT power diagram for the highlighted WASO period (bottom right) shows the two distinct frequency peaks that correspond to the alpha and ACA activities.

**Figure 2.**
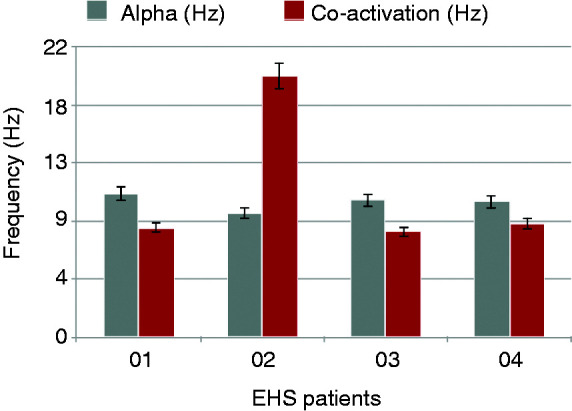
Bar plot of peak frequency values for alpha and ACA periods across the EHS group.

**Figure 3.**
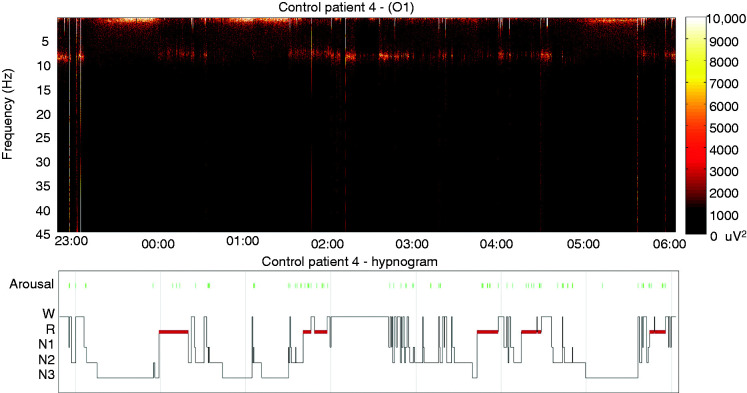
Representative whole-night EEG time-frequency analysis and hypnogram for a control patient. No ACA or other activities accompany alpha rhythm periods.

Five EHS patients (three females) aged between 53 and 69 years (mean age ± SEM: 58.2 ± 5) and 10 control subjects (58.1 ± 4) were identified (Tables 1 and 2). All EHS patients demonstrated oscillatory activity during pre-sleep wake or WASO periods that was additional to the expected alpha rhythm, and which we define here as “alpha co-activation” (ACA) (Figure 1). Specifically, in four EHS patients, ACA occurred at high theta/low alpha frequencies (8.39 ± 0.35 Hz; Table 1, Figure 2). The spectral profile of the ACA for one patient was found at beta frequencies (19.8 Hz). No ACA periods were present in pre-sleep wake, WASO or any other alpha rhythm periods of the matched control patients (Figure 3). To account for any additional “prodromal or premonitory” EEG fingerprint predating WASO periods, an event-related spectral perturbation analysis in relation to ACA was performed. However, we were unable to demonstrate any other dynamic EEG traces that preceded sleep/wake transitions and activation of ACA at the scalp sensor level.

**Table 1.** Demographic and sleep parameters of patients.

Patients	Gender	Age	WASO (min)	REM (%)	N1 (%)	N2 (%)	N3 (%)	Alpha peak (Hz)	Co-activation (Hz)
1	F	65	172.5	13.5	9.8	48.5	28.1	10.9	8.3
2	F	53	59.2	15.7	4.3	48.8	31.2	9.4	19.8*
3	M	69	164.8	19.7	8.4	51.1	20.7	10.4	8
4	F	63	68.9	24.2	14.3	35.9	22.1	10.3	8.6
5	M	41	56.4	19.1	15.3	46	19.5	9.87	8.64
Average		58.2	104.4	18.4	10.4	46.1	24.3	10.2	8.39
SD		11.3	58.9	4.1	4.5	6.0	5.1	0.6	0.3
SEM		5.0	26.4	1.8	2.0	2.7	2.3	0.3	0.1

Abbreviations: N1: non-REM stage 1; N2: non-REM stage 2; N3: non-REM stage 3; F: female; M: male; REM: rapid eye movement; SD: standard deviation.

* Excluded from average and standard deviation (SD) calculation.

**Table 2.** Demographic and sleep parameters of study controls.

Controls	Gender	Age	WASO (min)	REM (%)	N1 (%)	N2 (%)	N3 (%)	Alpha peak (Hz)
1	F	73	184.1	0	16.2	74	9.8	9.7
2	F	56	103.5	15.1	8.7	58.6	17.6	9
3	M	57	94.9	22.3	7.8	33.1	36.8	8.3
4	F	51	123.6	28.9	34	23.5	13.6	9.4
5	F	71	91.1	18.1	8.8	46.4	26.7	9.36
6	F	69	60.5	27.8	5.8	44.6	21.8	10.26
7	F	73	158.8	17.4	8.2	40.1	34.2	9.6
8	M	48	177.1	10	17.3	33.9	38.8	10.32
9	M	39	83.5	26.4	13	40.9	19.6	10.53
10	M	44	58.5	15.1	9.3	50.8	24.9	10.92
Average		58.1	113.6	18.1	12.9	44.6	24.4	9.5
SD		12.7	45.8	8.9	8.3	14.3	9.8	0.7
SEM		4.0	14.5	2.8	2.6	4.5	3.1	0.2

Abbreviations: N1: non-REM stage 1; N2: non-REM stage 2; N3: non-REM stage 3; F: female; M: male; REM: rapid eye movement; SD: standard deviation.

**Figure 4.**
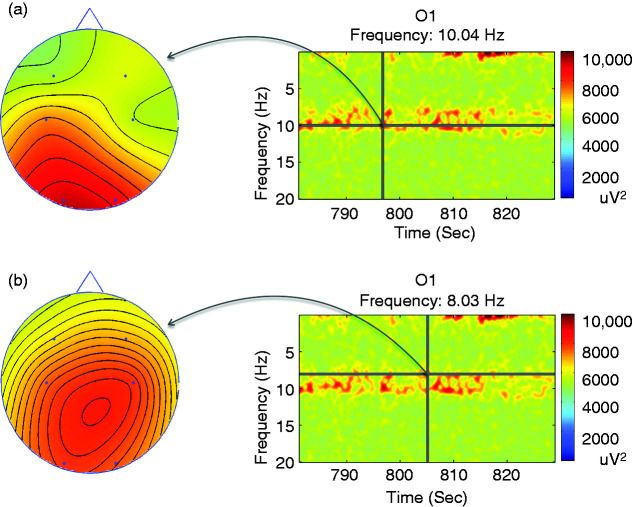
Spectral topography analysis showing distribution of power at sensor space for alpha (a) and ACA (b) bands during the second WASO period, as highlighted in Figure 1. It is evident that the targeted elements (right) for the characteristic alpha and ACA frequencies take maximal values at occipital and central-parietal areas, respectively.

Further analysis suggested that ACA and alpha rhythms differed in regard to their EEG topography. Specifically, spectral topography demonstrated ACA periods being primarily active, in terms of maximal power in the frequency domain, in centroparietal regions. This was in contrast to observed alpha rhythm, which predominated in occipital areas (Figure 4). Moreover, a higher order interaction between these two frequency bands was also observed during the TFA analyses. For example, a weaker ACA activity was concomitantly demonstrated in the occipital regions, mirroring the alpha rhythm peak. Similarly, some weak alpha rhythm was noted in the centroparietal regions, mirroring the concomitant ACA peak. We observed no such mirroring, or the double peaked profile, in controls.

**Figure 5.**
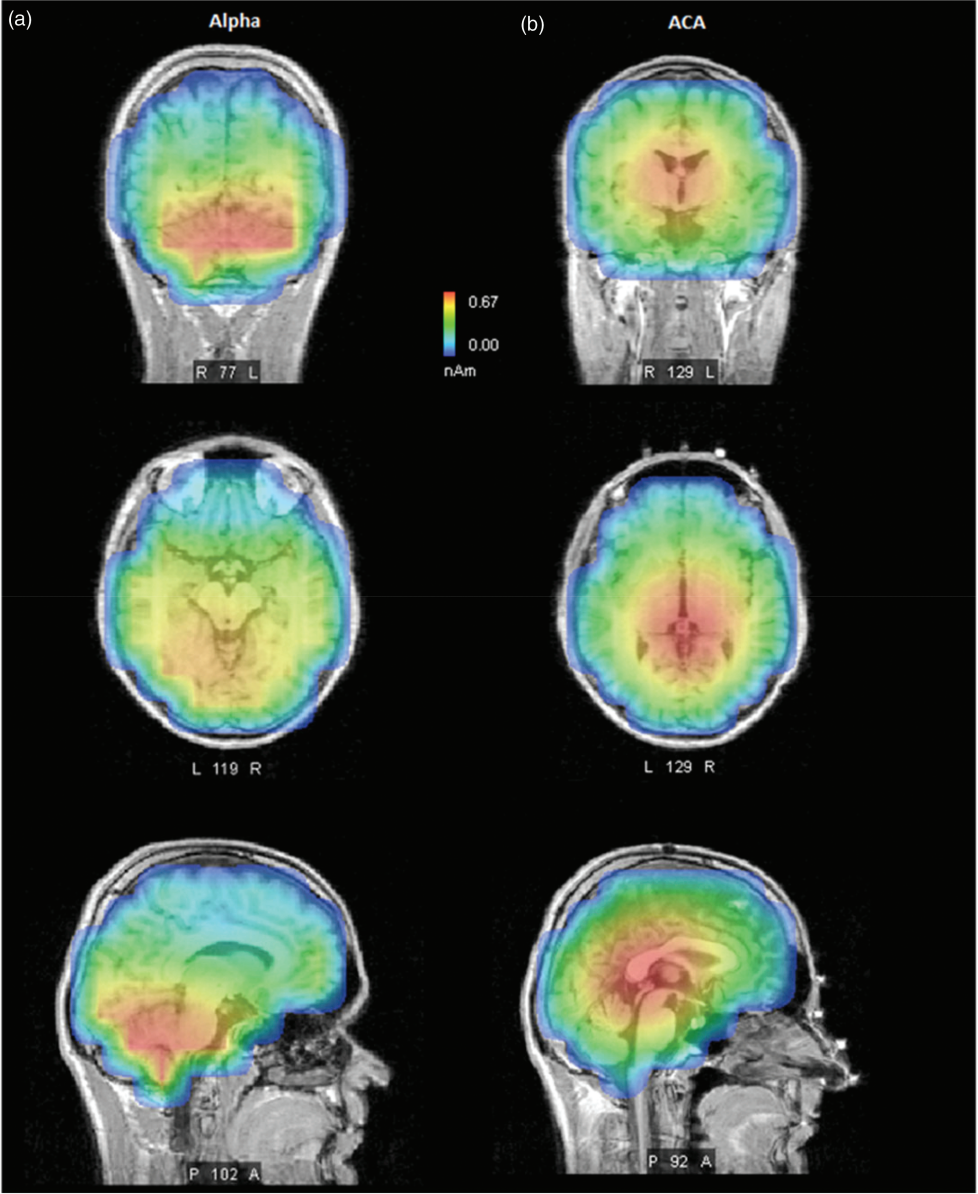
Representative source localisation analysis of a patient with EHS showing the origin of peaks at sensor space for alpha (a) and ACA (b) bands during the second WASO period. The alpha peak, reconstructed from signals that were band pass filtered from 8.5–12.5 Hz, appears to originate in the occipital region while the ACA peak, reconstructed from signals that were band pass filtered from 3.55–8.5 Hz, appears to originate in the deeper thalamic and centro-parietal region.

**Figure 6.**
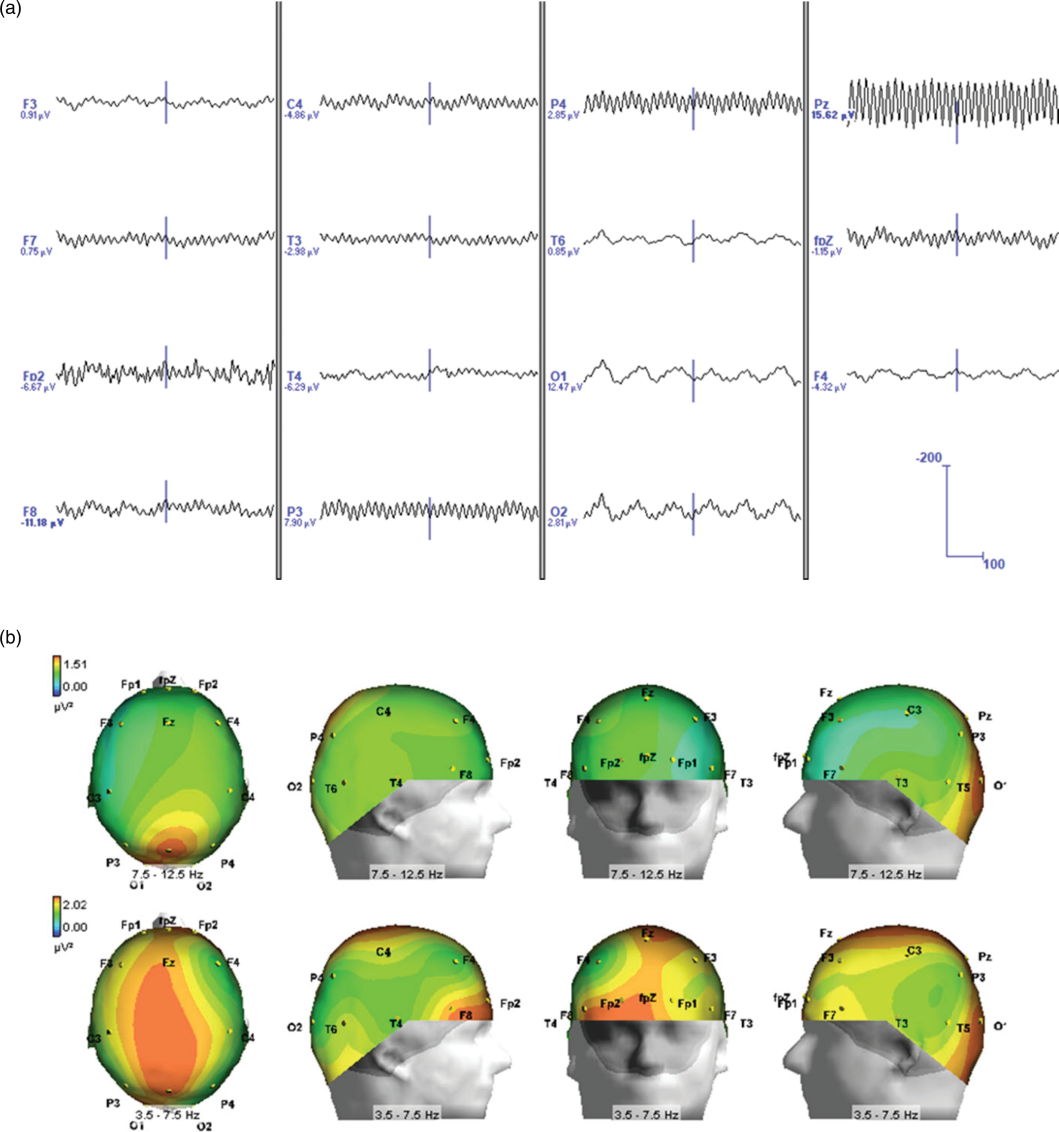
Temporal course of co-activation showing the concomitant alpha and ACA bands during the representative second WASO period of an EHS patient, band pass filtered to 3.5–12.5 Hz (a) and their 3D topography (b).

Source reconstruction analysis confirmed the spectral topography findings. Specifically, origins of the alpha activity were traced to the bilateral occipital cortices (Figure 5). Equally, the source of ACA activity was demonstrated as strongest in mesial structures (e.g. thalamic regions and in the posterior cingulate cortices, Figures 4–6). Of note, overall alpha activity in EHS patients was not shown to differ from those of controls, as judged by its peak frequency or its topography (Table 1 and 2; Figures 4–6). Thus, we suggest that ACA peaks, observed during the period of sleep-wake transition, are potential interictal biomarkers that showcase neurophysiological differences between EHS patients and controls.

## Discussion

Exploding head syndrome is an unusual phenomenon, although perhaps not as rare as previously thought. Some patients are referred to headache clinics, often with misperception and anxiety, shared by patients and healthcare professionals alike, that the symptoms may result from some sort of intracranial neurovascular disorder. This misbelief may be driven by the equally dramatic nomenclature of other headache phenotypes, such as ‘thunderclap headache’, which result from potentially life-threatening underlying pathology, such as subarachnoid haemorrhage. Thankfully, however, EHS may be regarded as a typically painless and benign sensory parasomnia, most often occurring at the wake-sleep transition (9).

The pathophysiological mechanisms underlying EHS have recently been the subject of renewed interest (9). This has undoubtedly been helped by an increasing recognition of a significant distress some of its sufferers seem to endure, highlighting the need for timely recognition and assurance, which is often only found after referral to secondary and tertiary care. Polysomnographic (PSG) recordings of individuals with EHS are rare and limited to small case series and individual reports (19–23). No reports of other electrophysiological investigations (for example auditory evoked potentials) exist. The findings of this small retrospective study are hence opportune, as they for the first time propose a novel EEG fingerprint potentially associated with this syndrome. We here report a putative EEG fingerprint of EHS in the form of an additional oscillatory activity of distinct topography (ACA, as described above), which takes place during alpha rhythm periods (WASO/pre-sleep wake) during the night (Figures 1–6). It is of note that in one small published series, authors similarly reported that captured events arose from drowsiness with predominant alpha rhythm interspersed with some theta activity (19). In one individual, EHS events occurred during the transition from wake to N1 sleep; in another they occurred in the reverse direction, and in the third patient arose during the transition from N2 sleep to wake (19). Arousals occurred in all recordings immediately following the event, and in no recording were epileptiform discharges identified from the scalp EEG (19). Arguably, these reports appear in striking keeping with our findings.

Notwithstanding EHS, sleep-wake transitions are already ordinarily characterised by a power increase in rhythms of various regions. The demonstration of additional ACA rhythms in EHS patients at those EEG hinterlands, which is absent in controls, can be taken to suggest abnormal simultaneous engagement of conflicting neurophysiological networks in afflicted patients, which could therefore be potentially viewed as an inherent (or inter – “ictal”) trait, or biomarker, the presence of which conveys the necessary neurophysiological substrate from which EHS attacks can manifest, perhaps under an additional set of circumstances or triggers that allow them to.

The historical view of alpha oscillations as an “idling” rhythm that characterises an alert but resting brain state (24) has long been challenged. An accumulating body of evidence points to neural correlates of consciousness (25) involving a subset of neurons engaged in alpha oscillations, which synchronise the sensori-fronto-parietal network, and that can, through their interactions, mediate top- down modulation (26). It has been argued that alpha frequency band oscillations contribute to recurrent processing and to top-down amplification (26–28). In addition, they have been shown to phase-lock between widely separated cortical regions (28). Phase synchrony has been shown as essential in the formation and communication of transient neuronal assemblies (29) and, in keeping, alpha oscillations have been argued as important in large-scale integration. Additionally, several studies demonstrated that alpha waves form functional large-scale networks during stimulus processing and task execution (26,28). Co-activation of other frequencies along with alpha has been reported to occur in response to cognitive demands (30), and the findings here may imply that contradictory cognitive processing may be taking place during transitional states of diminished consciousness in EHS. Of note, our pilot findings suggest deeper and posterior structures, such as thalamic pulvinar, as the source of ACA rhythm. In keeping with ACA rhythms’ hypothesised role in EHS, the thalamic pulvinar nucleus was recently demonstrated to participate in modulation of alpha synchrony between widespread cortical areas as a function of attentional demands (30). Moreover, Saalman et al. (2012) hypothesised a critical role for the thalamus not only in attentional selection but more generally in regulating information transmission across the visual cortex.

Recollection of contextual information represents the core of human recognition memory and it has been associated with increased theta power and interregional phase synchronization, especially relating to the septo-hippocampal network (27). Arguably, any erroneous pathophysiologic mechanisms in EHS would need to include aberrant recollection of contextual information. For example, for EHS patients to experience and report a true sensory episode of sudden loud noise, or to report a sense of explosion that may sometimes be accompanied by breathing irregularities and/or intense light flashes, one can surmise that any erroneous physiologic mechanisms would need to extend from a mere stimulus familiarity misrecognition. Given that theta rhythmicity recorded in the posterior cingulate cortex is thought to have a septo-hippocampal origin (31), perhaps it should not be surprising that source analyses of our EHS patient cohort, unlike that of controls, also strongly implicated the region of the posterior cingulate cortex in ACA generation in EHS. Finally, in potential further note to our findings, it has been recently proposed that confusional and psychotic states encountered in the PD-dementia with Lewy bodies might be a reflection of a thalamic dysfunction promoting a theta burst mode and the resulting thalamocortical dysrhythmia (32). Perhaps in the more global mechanistic mirage to the findings here, it has been proposed that the resulting thalamocortical dysrhythmia may act to inhibit the frontal attentional network and favor the decoupling of the default mode network, enabling fluctuating production of hallucinations and delusions in affected patients (32). Taken together, our findings offer conceivable, if very preliminary, support to the hypothesis that in EHS patients, at times of sleep-wake, transition aberrant attentional processing may lead to amplification and modulation of external sensory (e.g. auditory or visual) stimuli.

Findings here, therefore, may be taken to suggest a novel biomarker of this parasomnia, as well as provide initial insight into its pathophysiology and offer some future research directions. The limitations of our study primarily include the tertiary nature of our database and the small number of EHS patients identified over the investigated period, which, however, is indicative of the syndrome’s apparent rarity and its underreporting in general. In addition, the retrospective and cross-sectional nature of our study means that our findings can only be taken to imply an association and can not be taken to suggest directionality or the causation. Moreover, the presence of similar co-activation abnormalities in other sleep disorders cannot be ruled out by these findings and needs to be further investigated. Finally, our inability to identify EEG records pertaining to manifest EHS events in our database prevent a pivotal comparison of our interictal findings with EEG traces of EHS-attack events that could provide further crucial insight to EHS pathophysiology. It is nonetheless hoped that these novel preliminary findings may serve to encourage future longitudinal EEG recordings, which may be needed to capture these rare events, due to the complex nature of the syndrome. In conclusion, despite the perceived rarity of EHS, a concerted multidisciplinary effort of headache and sleep specialists might enable pivotal prospective multimodal imaging studies, conducted with a larger number of subjects able to authoritatively address any aberrant EHS pathophysiology beyond the scopes of this pilot investigation.

## Clinical implications

- Exploding head syndrome is an unusual, painless sensory parasomnia, commonly initially encountered in headache clinics, most often occurring at the wake-sleep transition.
- There is increasing recognition of a significant distress some of its sufferers may endure, highlighting the need for timely recognition and assurance, often only found after referral to secondary and tertiary care.
- The findings of this small retrospective study propose a novel EEG fingerprint potentially associated with this syndrome.
- We hypothesise that at times of sleep-wake transition in patients with exploding head syndrome, aberrant attentional processing may lead to amplification and modulation of external sensory stimuli.
